# A systematic review of pregnancy-related clinical intervention of drug regimens due to pharmacokinetic reasons

**DOI:** 10.3389/fmed.2023.1241456

**Published:** 2023-11-29

**Authors:** Lauren A. Borda, Mats Någård, David W. Boulton, Raman Venkataramanan, Paola Coppola

**Affiliations:** ^1^School of Pharmacy, University of Pittsburgh, Pittsburgh, PA, United States; ^2^Clinical Pharmacology and Quantitative Pharmacology, Clinical Pharmacology and Safety Sciences, R&D, AstraZeneca, Gaithersburg, MD, United States; ^3^Clinical Pharmacology and Quantitative Pharmacology, Clinical Pharmacology and Safety Sciences, R&D, AstraZeneca, Cambridge, United Kingdom

**Keywords:** pregnancy, pregnant, dose adjustment, contraindicated, pharmacokinetics

## Abstract

**Background and objective:**

Published works have discussed the pharmacokinetic interactions of drugs with pregnancy, but none comprehensively identify all the approved United States Food and Drug Administration (FDA) and European Medicines Administration (EMA) drugs that have a pregnancy-related intervention. The objective of this systematic review is to comprehensively identify medications that have clinically meaningful interventions due to pharmacokinetic reasons.

**Methods:**

An in-depth search of clinical data using the PDR3D: Reed Tech Navigator™ for Drug Labels was conducted from 1 June to 12 August 2022. The PDR3D was analyzed using the search terms “pregnant” and “pregnancy” within the proper label section. Regarding the US labels, the terms were searched under the “dosage and administration” section, whereas with the EU labels, the terms were searched within the “posology and method of administration” section. If a finding was discovered within the search, the rest of the label was analyzed for further information. Clinical relevance was based on whether an intervention was needed.

**Results:**

Using the search strategy, 139 US and 20 EU medications were found to have clinically meaningful interventions in pregnancy. The most common explanations for clinical relevance included hepatic metabolism, protein binding, renal elimination, and P-gp influence. Of the US labels: 40 were found to undergo hepatic metabolism, 11 were found to be influenced by renal elimination, 12 were found to be influenced by protein binding, 7 were found to be influenced by P-gp, and the remaining drugs required further research. Of the EU labels: 11 were found to undergo hepatic metabolism, 3 were found to be influenced by renal elimination, 3 were found to be influenced by protein binding, 1 was found to be influenced by P-gp, and the remaining drugs required further research.

**Conclusion:**

This comprehensive review of clinically relevant interventions in pregnancy will potentially aid in the treatment of pregnant females when they are undergoing therapy, provide intervention and dosing guidance for physicians, and save time for prescribers and pharmacists. Advances in non-clinical predictions for pregnancy dosing may guide the need for a future clinical evaluation.

## Introduction

Pregnant females are commonly prescribed medications for both pregnancy and non-pregnancy related disease states (1). Pregnancy does not exclude the need for pharmacotherapy, and proper control over a disease-state is essential, in some cases, to preventing poor fetal outcomes and the health of the females. In the United States (US), over 50% of pregnant females take at least one medication, and the national average ranges from 3 to 5 medications per pregnant female (2). The dosing of medications during pregnancy is more difficult to predict compared to a non-pregnant female because of the extensive anatomical and physiological changes that a female will undergo throughout all three trimesters (1). Unfortunately, the dosing regimens in pregnancy for many medications are both widely unknown and understudied. Physicians are dealing with limited knowledge in the pregnancy population and will often prescribe medications where the dose may not be adequate and has not been studied (1). This is especially true in the case of older drugs that have an established history of use in pregnancy. Without the established dosing regimens, the safety of both the pregnant female and the fetus may be at risk. Recently, the United States Food and Drug Administration (FDA) has been focusing on increasing research into pregnancy to provide comprehensive guidelines for use of medications and prevent adverse outcomes (1). In doing this, the FDA has increased pregnancy investigation requirements, provided in-depth recommendations on how to integrate pregnant females in clinical trials, and replaced the five-letter risk categories with extensive risk information in prescribing information. If this trend were to continue, it would be beneficial to have a comprehensive list of drugs to establish both the presence and lack of clinically meaningful intervention information that is currently available.

This systematic review is intended to identify the drugs that have been labeled for pregnancy-related clinical intervention due to pharmacokinetic reasons by the FDA and European Medicines Agency (EMA). Drugs that are contraindicated in pregnancy due to fetal toxicity are not considered. This clinically relevant data provides aid for dosing guidance for prescribers, mechanisms of altered disposition where they are known, as well as identification of the need for more research into establishing safe and effective dosing in pregnant females.

## Mechanisms for altered drug disposition in pregnancy

### Absorption

Absorption is the movement of drug from the route of administration into the body (2). Absorption can be characterized using bioavailability, which is the fraction of parent or active drug that reaches systemic circulation. When medications are administered orally, the bioavailability can greatly vary due to factors such as gastric pH and gastrointestinal transit time. During pregnancy, increasing concentrations of progesterone will delay gastric emptying, and the increase in production of gastrin by the placenta will lead to a reduction in gastric pH (3). Consequently, these factors can result in altered drug bioavailability, particularly decreasing the absorption of weak acids, as well as a delayed time to peak systemic concentrations after administration (4). Intestinal transporters, like P-glycoprotein (P-gp) or breast cancer resistance protein (BCRP), can also affect drug absorption from the gastrointestinal tract due to increased efflux activity in pregnancy, which effectively reduces the bioavailability of some drugs (5).

### Distribution

Distribution is the transport of drug between body compartments (1). The pharmacokinetic parameter of volume of distribution (Vd) can be described as the extent that a drug is dispersed throughout the body (2). Distribution can be influenced by factors such as changes in organ blood flow, concentrations of plasma proteins, amounts of total body water, and fat mass (4). During pregnancy, an increased amount of extracellular fluid may translate to lower peak and steady-state systemic concentrations for hydrophilic drugs, whereas an accumulation of fat tissue may lead to lower systemic concentrations for hydrophobic drugs (6). Additionally, reduced concentrations of plasma proteins in pregnancy may increase the free fraction of highly protein-bound drugs, which may increase the distribution of drug into the tissues (5).

### Metabolism

Metabolism can be described as the process by which drugs are modified via enzymes, to yield metabolites that can be more easily eliminated from the body through urine and/or feces (2). Drug metabolism is split into 2 categories: phase 1 metabolism includes the oxidation, reduction, and hydrolysis reactions whereas phase 2 metabolism are conjugation reactions. Phase 1 reactions are primarily performed by the cytochrome P450 (CYP) family of enzymes, and phase 2 is primarily performed by transferases such as uridine 5′ diphosphate glucuronosyltransferases (UGTs). Pregnancy can alter the expression of enzymes in both phase 1 and phase 2 metabolism leading to increased or decreased activity. For example, the activities of CYP3A4 (7), CYP2B6 (8), CYP2D6 (9), and CYP2C9 (10) are increased in pregnancy, whereas the activities of CYP1A2 (11) and CYP2C19 (10) are decreased. The expression of transferases has not been well studied, but it is known that the activity of UGT1A4 is increased (12), UGT1A1 is increased (13), and UGT2B7 does not change (14). These alterations in enzyme activity may change drug exposure within the body (4).

### Excretion

Excretion is the process of clearing administered drug and metabolites from the body via the urine and feces (2). There are many factors that contribute to the excretion of a drug and its metabolites. In pregnancy, there is a decrease in the excretion of some lipid-soluble drugs due to increased fat mass, and a general increase in the excretion of some drugs due to increased cardiac output (2). Additionally, the renal blood flow will increase in pregnancy, which leads to increased renal clearance (4). Elevations in renal clearance in pregnancy may also be explained by increased renal transporter activity of organic cation transporter 2 (OCT2), multidrug and toxic compound extrusion transporter 1 (MATE1) and MATE2-K (15).

It is important to recognize that pregnancy is not a single pharmacokinetic state, rather a changing state, including the post-natal phase, before returning to a “non-pregnant” state. The significant and complex changes described above in the absorption, distribution, metabolism, and excretion of medications during pregnancy can alter the systemic exposure of the drug and how a drug will act within the body. The changes may vary by the stage of the pregnancy and in some cases concomitant mechanisms that have opposite effects may result in no or small changes in systemic exposure.

## Methods

This review is a Preferred Reporting Items for Systematic Reviews and Meta-Analyses (PRISMA) 2020-guided systematic review to identify medications that have clinically relevant intervention data when used during pregnancy. The search strategy was completed using the PDR3D: Reed Tech Navigator™ for Drug Labels, which is a database that contains US prescribing information (USPI) and summary of product characteristics (SmPC) for approved medications by the FDA and EMA. For the USPI the terms “pregnant” and “pregnancy” were searched within the “dosage and administration” section. For the SmPC, the terms “pregnant” and “pregnancy” were searched within the “posology and method of administration” section. If a finding was discovered within the search, the rest of the label was analyzed for further information. The PDR3D database was searched during the period of 1 June 2022–12 August 2022.

The information collected from the database was organized based on the presence of clinically relevant interventions for pregnancy. Clinical relevance was assessed based on if there was a recommendation for use in pregnancy highlighted in the prescribing information. This could be a dose adjustment, lack of a dose adjustment, contraindication, or the recommendation to ask a health professional before use.

Throughout the search strategy described above, repeat medications, treatment for infertility, contraceptives, supplements/vitamins, devices, duplicate records among dosage forms and non-FDA or EMA approved medications were removed. The remaining medications were assessed for clinically meaningful interventions that were advised by the USPI or the SmPC. Those with clinically meaningful interventions were then evaluated for a description of the mechanism(s) of change. Many of the mechanism(s) of change could not be found within the prescribing information and therefore PubMed was used for further research to determine the likely mechanism(s) or pharmacokinetic reasoning behind the clinical intervention. Any recommendation of use within this comprehensive review was taken from the USPI and SmPC.

## Results

The Supplementary tables described below can be utilized to examine the results of the in-depth search of clinical data and list the findings for each individual medication.

**FIGURE 1:**
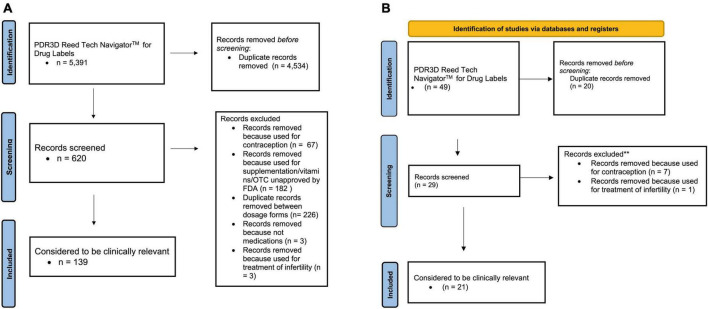
**(A)** PRISMA 2020 flow diagram for updated systematic reviews for US FDA labels for pharmacokinetic changes in pregnancy. **(B)** PRISMA 2020 flow diagram for updated systematic reviews for EMA labels for pharmacokinetic changes in pregnancy.

Figures 1A, B display the PRISMA flow diagram of the search strategy, exclusions and output that was further assessed with PubMed for US FDA and EU labels, respectively. PDR3D was used for “identification” purposes, and “screening” excluded contraceptives, infertility treatments, repeat medications, devices, supplements/vitamins/unapproved by the FDA and EMA, and duplicate records between dosage forms.

In this comprehensive review, 139 US and 20 EU medications were found to have clinically meaningful interventions in pregnancy that were captured in labeling. Medications that have been labeled for pregnancy-related clinical intervention due to pharmacokinetic changes are shown in Supplementary Table 1. Supplementary Table 1 expands upon each mechanism of change, pharmacokinetic data, clinical data, and references. Supplementary Table 1A refers to the US labels, whereas Supplementary Table 1B refers to the EU labels.

Supplementary Table 2 lists the medications that included the recommendation “ask a health professional before use” or “contraindicated” but upon further investigation there is literature to potentially suggest pharmacokinetic data that would support dose adjustments in pregnancy that is not currently supported by labeling. Supplementary Table 2 refers to the US labels. There were no EU labels that fit these criteria.

Supplementary Table 3 lists the medications that included the recommendation “ask a health professional before use” or “contraindicated” but upon further investigation there is no adequate literature that we identified to potentially suggest pharmacokinetic data that would support dose adjustments in pregnancy based on safety concerns. Supplementary Table 3A refers to the US labels, whereas Supplementary Table 3B refers to the EU labels.

**FIGURE 2:**
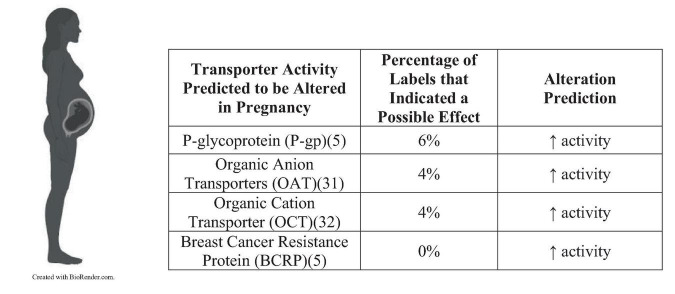
Percentage of US FDA labels that described pregnancy-related changes in transporter activity.

The most common explanations for clinically relevant intervention included altered metabolism, protein binding, renal clearance, and P-gp activity. Of the US labels: 40 had altered metabolism, 11 were found to be influenced by renal clearance, 12 were found to be influenced by protein binding, 10 were found to be influenced by drug transporters, and the remaining drugs required further research into the underlying mechanism(s). Of the EU labels: 11 had altered metabolism, 3 were found to be influenced by renal clearance, 3 were found to be influenced by protein binding, 1 was found to be influenced by drug transporters, and the remaining drugs required further research into the underlying mechanism(s). Figure 2 summarizes the percentage of US FDA labels that described pregnancy-related changes in transporter activity. No EU labels described transporter effects.

## Discussion

The resources available to healthcare professionals permit the identification of medications that may require clinical intervention with pregnancy, but a comprehensive review of this topic requires the use of multiple sources which becomes challenging in clinical practice. A single resource may aid in the optimal treatment of pregnant females who are undergoing pharmacological therapy.

Pregnancy affects a number of important metabolic pathways, and therefore many drugs will have different systemic exposures during pregnancy versus a non-pregnant female (2). Metabolism was the most common reason for clinical intervention because of pregnancy. The influence of pregnancy on hepatic clearance of drugs differs by enzymatic pathway and pregnancy can alter metabolizing enzymes by either increasing or decreasing their activities. Many drugs are known to undergo multiple pathways of metabolism, and therefore it is difficult to predict exactly how a drug will be affected by pregnancy. Not all drugs within a class can be viewed as having similar changes in pregnancy due to metabolic changes. For example, most antiretroviral drugs are known to be primarily metabolized via CYP3A4. When drugs primarily undergo metabolism via CYP3A4, it can be expected that a lower systemic exposure of the drug will occur within a pregnant female due to increased activity of CYP3A4. Antiretroviral drugs like atazanavir and darunavir have lower exposure during pregnancy (16, 17). Studies have shown the CYP3A4 substrate atazanavir AUC was lower during pregnancy versus HIV-infected non-pregnant patients (18), and a 25% reduction in atazanavir exposure was reported when compared to postpartum (19). The recommended pregnancy dosing is atazanavir 300 mg with ritonavir 100 mg once daily with food, with dosing modifications for some concomitant medications (20), which is not a dose adjustment when compared to non-pregnant adults. The EMA also recommends therapeutic drug monitoring for atazanavir in pregnancy as a precaution for the potential risk of insufficient exposure (21). A population PK analysis showed the CYP3A4 substrate darunavir exposure was 24 and 23% lower in pregnancy for standard once daily and twice daily, respectively, and the probability of maintaining therapeutic exposure during pregnancy was higher for standard twice daily dosing compared to once daily dosing (16). By contrast, etravirine is metabolized by CYP3A4, but it is also metabolized by CYP2C9 and CYP2C19 (22). Etravirine’s systemic exposure during the third trimester of pregnancy increases, likely due to decreased CYP2C19 activity, which counterbalances the increased CYP3A4 activity. The overall change in systemic exposure is not considered to be clinically relevant and no dosage adjustment of etravirine in pregnancy is recommended (22). It should be noted that the concentration changes of a drug used to suppress the viral load of HIV does not necessarily need to be dose-adjusted unless it is no longer adequately suppressing the viral load within the pregnant patient (23). The recommended HIV viral load that is considered to be suppressed is < 50 HIV RNA copies/mL.

Increases in plasma volume during pregnancy results in a rise in the glomerular filtration rate (GFR) (2). There is approximately a 50% increase in GFR during the first trimester and it will continue to rise throughout the next two trimesters (5). Lithium is primarily cleared via the kidneys and the blood concentrations of lithium is expected to decrease during pregnancy (24). Throughout the pregnancy lithium blood concentrations have been shown to decrease by 13 to 47% (25). The FDA recommends to avoid sodium restriction and diuretics to maintain lithium’s therapeutic window (26). At delivery, the vascular volume will rapidly decrease, and lithium clearance can decline to pre-pregnancy systemic exposures. Therefore, the FDA recommends to decrease or discontinue lithium therapy 2–3 days before the expected delivery date to reduce the risk of lithium intoxication.

It is important to note that a drug being eliminated via renal excretion does not necessarily mean that a dose adjustment will be needed. Within this search, the renally eliminated drugs were often eliminated by multiple pathways, so dose adjustments were not necessary in most cases.

Increasing plasma volumes in pregnant females will cause concentrations of albumin and α1-acid glycoprotein (AAG) to decline by 20 to 40% by the third trimester (2). When plasma protein concentrations decrease, it leads to a decrease in drug binding to plasma proteins. This causes an increase in unbound drug concentrations and may lead to increased clearance of some drugs. Serum concentrations of phenytoin are predicted to fall by approximately 60% in the third trimester (27). Phenytoin has a narrow therapeutic index (28) and is highly protein bound with a low clearance (29). During pregnancy, decreased exposure of phenytoin can occur likely because of increased unbound fraction and increased clearance. The USPI recommends that periodic measurements of unbound serum concentrations in pregnant females should be performed during treatment, and the dose should be adjusted as necessary. Within this review, there were several researchers that speculated that an increase in unbound concentrations in pregnancy may have increased the clearance of a number of drugs that had decreased serum concentrations. In these cases, most of the researchers did not measure the unbound concentrations and therefore could not make a comprehensive evaluation. It is recommended to study the free fraction of drugs when characterizing their pharmacokinetics in pregnant females.

Drug transporters can be expressed within a variety of organ systems, and they can affect pharmacokinetics of a drug if it is a substrate (5). For example, intestinal luminal transporters affect drug absorption from the gastrointestinal tract, hepatic sinusoids control drug uptake into hepatocytes for metabolism, and transporters expressed in the renal cells control reabsorption and tubular secretion. Major drug transporters that are predicted to be altered during pregnancy include P-gp, organic anion transporters (OATs), OCTs, and BCRP (30–32). P-gp is a transporter that acts at multiple sites, including the intestines, hepatocytes, kidney proximal tubules, brain endothelial cells, and placenta (5). If the activity of P-gp is altered, then it will influence the absorption, excretion, and extent of drug transport into target organ systems. P-gp works as an efflux transporter, so an induction of activity will cause there to be less bioavailability if it is a substrate of this transporter. Researchers have assumed that drug transporter expression is altered during pregnancy, but most transporters are not comprehensively studied. Despite a lack of knowledge into pregnancy’s influence over transporters, 6% of the 139 US drug labels were likely influenced by P-gp, 4% to OATs, and 4% to OCTs. More research needs to be focused toward the effects of these transporters because they will likely influence bioavailability and clearance during pregnancy.

Considering the complexities of the three proposed most common mechanisms of drug concentration differentiation, it is paramount to discuss when all three mechanisms coincide. Within this comprehensive search, methadone stood out as having multiple possible explanations for decreased serum concentrations in pregnant females. Methadone is highly metabolized by CYP2B6 and CYP3A4, highly eliminated via renal excretion, and is highly protein bound (33). Methadone is also a substrate of P-gp, but there is a lack of research or hypotheses into how being a P-gp substrate may impact methadone’s exposure in pregnancy (34). Methadone clearance increases throughout pregnancy, and it parallels decreased serum trough concentrations (33). It is recommended that a female’s methadone dose either be increased or the dosing interval decreased. The best method has yet to be established, and there is insufficient evidence to suggest a routine measurement of methadone concentrations.

Treatments for human immunodeficiency virus (HIV) have been highly studied in pregnancy. Cobicistat is a pharmacokinetic booster that is co-administered with antiretrovirals to boost the antiretroviral plasma concentrations (35). Cobicistat inhibits the metabolism of the antiretroviral via CYP3A4, which increases their plasma concentrations and prolongs half-life. Cobicistat itself is also metabolized by CYP3A4. Pregnancy increases the activity of CYP3A4, and cobicistat’s systemic exposure decreases during pregnancy, which decreases its effectiveness as a booster. Due to the risk of treatment failure and perinatal transmission of HIV, in 2018 the FDA chose to change the cobicistat-containing product labels to being not recommended in pregnancy. Products containing cobicistat were available for use in pregnant females for 6 years before the FDA changed the labeling (36). Another pharmacokinetic booster is ritonavir, which is also a CYP3A inhibitor and a substrate, but which is currently approved for use in pregnancy for both for US and EU. Ritonavir’s systemic exposure is reduced by 30–50% in the second and third trimester (17). The USPI recommends increasing the dosing frequency of ritonavir and the corresponding antiretroviral being administered from once daily to twice daily. It is not recommended to increase the ritonavir dose because of the lack of evidence and concerns about tolerability in pregnant females.

The evaluation of the impact of pregnancy on drug disposition is often understudied and introduces many ethical challenges. Unfamiliarity with treatment regimens and the lack of comprehensive data often leads physicians to rely on their own clinical experience rather than being supported by prescribing information (1). Whilst analyzing prescription labels, it was found that certain selective serotonin reuptake inhibitors (SSRIs) and serotonin and norepinephrine reuptake inhibitors (SNRIs) had specific recommendations for use in pregnancy that were altered in recent years. As an example, in 2010, paroxetine’s prescribing information stated that “the physician may consider tapering paroxetine in the third trimester (37).” Most antidepressants had this recommendation because it was thought that neonates exposed to those medications late in the third trimester have developed complications at birth, such as persistent pulmonary hypertension of the newborn.

These recommendations were challenged soon after they were released by the FDA in 2004 (38). Researchers believed that the recommendations were based on a small amount evidence that could put both the mother and baby at risk. Particularly, concerning the dangers of untreated pre- and post-partum depression. In 2010, there was a study performed where it was examined whether discontinuing the SSRI 14 days before delivery would reduce the rate of neonatal symptoms (39). It was found that there were no differences between the neonates once the study and control groups were controlled for maternal illness severity. Presently, the prescribing information for SSRIs and SNRIs no longer recommend tapering in the third trimester. It was not well documented when this change took place, but by 2014 there was no longer a tapering recommendation for the third trimester in the paroxetine prescribing information. The prescribing information currently states, “When treating a pregnant female with paroxetine, the physician should carefully consider both the potential risks of taking an SSRI, along with the established benefits of treating depression with an antidepressant. This decision can only be made on a case-by-case basis (40).” SSRI and SNRI products were available for use in pregnant females for at least 10 years before the FDA decided to exclude the tapering recommendation. Additionally, pharmacokinetic studies of SSRIs and SNRIs have found that, depending on the medication, a dose adjustment may be necessary during pregnancy due to changes in exposure. To reuse the example of paroxetine, decreases in serum concentrations of paroxetine during pregnancy have been described (41). The authors concluded that this may necessitate an 100% increase in dose during the 3rd trimester to maintain concentrations. This suggests that tapering during the third trimester could result in subtherapeutic serum concentrations and put both the mother and possibly the fetus at risk.

Pregnancy influences many physiological functions, and it can increase demands for certain hormones that are necessary for both the mother and fetus’ health. For females who do not produce these hormones effectively or at all, it may influence their dosing regimens once pregnant. For example, hypothyroidism is a disease-state where the thyroid gland does not produce a sufficient amount of thyroid hormones, and a female needs higher concentrations of thyroid hormones during pregnancy (42). In females with normal thyroid functionality, the thyroid gland will enlarge and increase production of hormones to meet the need during pregnancy. The fetus will not begin to make enough thyroid hormone on its own until 18 to 20 weeks of pregnancy (43). If a female with hypothyroidism becomes pregnant, their dose of levothyroxine (T4) or liothyronine (T3) needs to be increased. Levothyroxine is the preferred choice for use in pregnant females since T4 can enter a fetus’ brain more readily than T3 (44). This is a unique example of the need for a dose adjustment because the alteration in dose is not a result of the pharmacokinetics of the drug changing during pregnancy, but there is simply an increased need for the fetus.

The ethics of testing drugs within pregnant females is a prominent issue for the scientific community. Including pregnant females in clinical trials would be beneficial for the future of gestational medicine, but ethical and safety implications should be considered in the mother-fetus benefit/risk evaluation. The use of pharmacokinetic modeling approaches could support the investigation of the expected drug systemic exposure in pregnancy. Physiologically-based pharmacokinetic (PBPK) models integrate both drug physiochemical properties and pregnancy-related physiological changes (1). PBPK can predict pharmacokinetic changes in pregnancy and provide a basis for supporting dose optimization for pregnant females in clinical trials. Overall, pharmacokinetic modeling can be a helpful tool to enable the inclusion of pregnant females in clinical trials with doses that are likely to be safe and effective.

In 2015, the FDA began to implement a pregnancy labeling system that required Sponsors to include all available data on the use of a medication within pregnancy. This information includes the risks of exposure to the fetus, rates of birth defects and miscarriage, the impacts of untreated disease and applicable dose adjustments, and studies that have been performed in both humans and animals. In general, the EMA prescribing information have similar data as their American counterpart. Whilst conducting this comprehensive review, within the parameters of our search, it was found that the FDA has identified a larger number of established dosing in pregnancy than the EMA. There were 139 USPIs were identified as containing recommendations for clinical action in pregnancy, and 40 of those were found to have established doses in pregnant females. There were 20 EMA SmPCs identified as containing recommendations for clinical action in pregnancy, and 8 were found to have established doses in pregnant females. One reason for the difference between the amount of established FDA and EMA recommendations in pregnancy may be the history of regulation in this area. Some of the FDA’s most relevant contributions in gestational health is the speed in which they established regulations in this area (45). In 2014, the FDA established requirements for pregnancy labeling, and within the next 2 years the US Department of Health and Human Services had established a task force on conducting research specific to pregnant females. In 2019, the FDA drafted guidance for industry on the scientific and ethical considerations for inclusion of pregnant females in clinical trials, as well as for post-approval pregnancy safety guidelines. Within 5 years, they had set up a comprehensive system to enable the creation of prescribing guidelines in pregnancy. Comparatively, the EMA approved guidelines on good pharmacovigilance practices within pregnant females in 2019. This difference in timing may explain the smaller number of established dosing for pregnant patients between the two regulatory bodies.

Limitations of this review are that the search strategy was not replicated by a second individual, the risk for incomplete retrieval of clinically relevant data via the search strategy used, and any additional data available after 1 August 2022 was not captured. Additionally, the Supplementary information provided in the review are derived from studies that utilized small sample sizes and inconsistent comparator groups. Many of the pharmacokinetic mechanistic explanations from the literature provided in this review have been inferred from the data but need further verification.

## Conclusion

This comprehensive review of clinically relevant interventions in pregnancy will potentially aid in the treatment of pregnant females when they are undergoing therapy, provide intervention and dosing guidance, and save time for prescribers and pharmacists. This review emphasized the importance of regulatory bodies recognizing the drastic changes that a female’s body will go through throughout pregnancy and the impact this has on the pharmacokinetics of many drugs. It is important to adjust the dose of affected drugs accordingly to ensure that they remain safe and effective. There is potential for improvement to be made with identification of dose adjustments during pregnancy but including the available risk information is helpful to clinicians who need to assess the risks and benefits of treatment. Additionally, clinically relevant data was gathered from the EMA SmPCs to better apply this review outside of the US. Dose adjustment in pregnancy is highly dependent on the individual drug’s physiochemical properties and the alterations of pharmacokinetics and physiological changes that occur during pregnancy. It is important for prescribers to be aware that individual drugs within a drug class cannot be prescribed in the same manner. Further research is required in this area, but dose adjustments should be guided based on the data available.

## Data availability statement

The original contributions presented in the study are included in the article/Supplementary material, further inquiries can be directed to the corresponding author.

## Author contributions

LB conducted the systematic review. All authors contributed to the analysis, interpretation, and conceptualization of the identified data; wrote various drafts of the manuscript; gave agreement to be accountable for all aspects of the work; and provided final approval of the version to be published.

## Supplementary material

Supplementary Table 1A Medications that have been labeled for pregnancy-related clinical intervention by the United States Food and Drug Administration.

Supplementary Table 1B Medications that have been labeled for pregnancy-related clinical intervention by the European Medicines Agency.

Supplementary Table 2 Medications that included the recommendation “ask a health professional before use” or “contraindicated” by the United States Food and Drug Administration that literature potentially suggests pharmacokinetic data that may support dose adjustments in pregnancy.

Supplementary Table 3A Medications that included the recommendation “ask a health professional before use” or “contraindicated” by the United States Food and Drug Administration where there is no clear literature data to indicate pharmacokinetic changes that would support dose adjustments in pregnancy.

Supplementary Table 3B Medications that included the recommendation “ask a health professional before use” or “contraindicated” by the United States Food and Drug Administration where there is no clear literature data to indicate pharmacokinetic changes that would support dose adjustments in pregnancy.

## References

[1] Abduljalil K Badhan R. Drug dosing during pregnancy-opportunities for physiologically based pharmacokinetic models. *J Pharmacokinet Pharmacodyn.* (2020) 47:319–40. 10.1007/s10928-020-09698-w 32592111

[2] Jeong H. Altered drug metabolism during pregnancy: hormonal regulation of drug-metabolizing enzymes. *Expert Opin Drug Metab Toxicol.* (2010) 6:689–99. 10.1517/17425251003677755 20367533 PMC3686288

[3] Tan E Tan E. Alterations in physiology and anatomy during pregnancy. *Best Pract Res Clin Obstet Gynaecol.* (2013) 27:791–802. 10.1016/j.bpobgyn.2013.08.001 24012425

[4] Pariente G Leibson T Carls A Adams-Webber T Ito S Koren G. Pregnancy-associated changes in pharmacokinetics: a systematic review. *PLoS Med.* (2016) 13:e1002160. 10.1371/journal.pmed.1002160 27802281 PMC5089741

[5] Feghali M Venkataramanan R Caritis S. Pharmacokinetics of drugs in pregnancy. *Semin Perinatol.* (2015) 39:512–9. 10.1053/j.semperi.2015.08.003 26452316 PMC4809631

[6] Krauer B Krauer F. Drug kinetics in pregnancy. *Clin Pharmacokinet.* (1977) 2:167–81. 10.2165/00003088-197702030-00002 328205

[7] Aweeka F Hu C Huang L Best B Stek A Lizak P Alteration in cytochrome P450 3A4 activity as measured by a urine cortisol assay in HIV-1-infected pregnant women and relationship to antiretroviral pharmacokinetics. *HIV Med.* (2015) 16:176–83. 10.1111/hiv.12195 25407158 PMC4320673

[8] Dickmann L Isoherranen N. Quantitative prediction of CYP2B6 induction by estradiol during pregnancy: potential explanation for increased methadone clearance during pregnancy. *Drug Metab Dispos.* (2013) 41:270–4. 10.1124/dmd.112.047118 22815312

[9] Wadelius M Darj E Frenne G Rane A. Induction of CYP2D6 in pregnancy. *Clin Pharmacol Ther.* (1997) 62:400–7. 10.1016/S0009-9236(97)90118-1 9357391

[10] Ke A Nallani S Zhao P Rostami-Hodjegan A Unadkat J. Expansion of a PBPK model to predict disposition in pregnant women of drugs cleared via multiple CYP enzymes, including CYP2B6, CYP2C9 and CYP2C19. *Br J Clin Pharmacol.* (2014) 77:554–70. 10.1111/bcp.12207 23834474 PMC4371535

[11] Tsutsumi K Kotegawa T Matsuki S Tanaka Y Ishii Y Kodama Y The effect of pregnancy on cytochrome P4501A2, xanthine oxidase, and N-acetyltransferase activities in humans. *Clin Pharmacol Ther.* (2001) 70:121–5. 10.1067/mcp.2001.116495 11503005

[12] Ohman I Luef G Tomson T. Effects of pregnancy and contraception on lamotrigine disposition: new insights through analysis of lamotrigine metabolites. *Seizure.* (2008) 17:199–202. 10.1016/j.seizure.2007.11.017 18201913

[13] Jeong H Choi S Song J Chen H Fischer J. Regulation of UDP-glucuronosyltransferase (UGT) 1A1 by progesterone and its impact on labetalol elimination. *Xenobiotica.* (2008) 38:62–75. 10.1080/00498250701744633 18098064 PMC3691104

[14] O’Sullivan M Boyer P Scott G Parks W Weller S Blum M The pharmacokinetics and safety of zidovudine in the third trimester of pregnancy for women infected with human immunodeficiency virus and their infants: phase I acquired immunodeficiency syndrome clinical trials group study (protocol 082). Zidovudine Collaborative Working Group. *Am J Obstet Gynecol.* (1993) 168:1510–6. 10.1016/S0002-9378(11)90791-1 8098905

[15] Bergagnini-Kolev M Hebert M Easterling T Lin Y. Pregnancy increases the renal secretion of N(1)-methylnicotinamide, an endogenous probe for renal cation transporters, in patients prescribed metformin. *Drug Metab Dispos.* (2017) 45:325–9. 10.1124/dmd.116.073841 28069720 PMC5325061

[16] Schalkwijk S Ter Heine R Colbers A Capparelli E Best B Cressey T Evaluating darunavir/ritonavir dosing regimens for HIV-positive pregnant women using semi-mechanistic pharmacokinetic modelling. *J Antimicrob Chemother.* (2019) 74:1348–56. 10.1093/jac/dky567 30715324 PMC6477987

[17] Salama E Eke A Best B Mirochnick M Momper J. Pharmacokinetic enhancement of HIV antiretroviral therapy during pregnancy. *J Clin Pharmacol.* (2020) 60:1537–50. 10.1002/jcph.1714 32798276 PMC8227837

[18] Conradie F Zorrilla C Josipovic D Botes M Osiyemi O Vandeloise E Safety and exposure of once-daily ritonavir-boosted atazanavir in HIV-infected pregnant women. *HIV Med.* (2011) 12:570–9. 10.1111/j.1468-1293.2011.00927.x 21569187

[19] Mirochnick M Best B Stek A Capparelli E Hu C Burchett S Atazanavir pharmacokinetics with and without tenofovir during pregnancy. *J Acquir Immune Defic Syndr.* (2011) 56:412–9. 10.1097/QAI.0b013e31820fd093 21283017 PMC3125419

[20] Atazanavir. *Atazanavir capsules 150 mg 60’s 115x40 mm (75cc).* Piscataway, NJ: Camber Pharmaceuticals, Inc (2022).

[21] Atazanavir Mylan. *Atazanavir Mylan | European Medicines Agency.* Mulhuddart: Mylan Pharmaceuticals Limited (2022).

[22] Mulligan N Schalkwijk S Best B Colbers A Wang J Capparelli E Etravirine pharmacokinetics in HIV-infected pregnant women. *Front Pharmacol.* (2016) 7:239. 10.3389/fphar.2016.00239 27540363 PMC4972814

[23] Boucoiran I Albert A Tulloch K Wagner E Pick N van Schalkwyk J Human immunodeficiency virus viral load rebound near delivery in previously suppressed, combination antiretroviral therapy-treated pregnant women. *Obstet Gynecol.* (2017) 130:497–501. 10.1097/AOG.0000000000002133 28796673

[24] Poels E Bijma H Galbally M Bergink V. Lithium during pregnancy and after delivery: a review. *Int J Bipolar Disord.* (2018) 6:26. 10.1186/s40345-018-0135-7 30506447 PMC6274637

[25] Wesseloo R Wierdsma A van Kamp I Munk-Olsen T Hoogendijk W Kushner S Lithium dosing strategies during pregnancy and the postpartum period. *Br J Psychiatry.* (2017) 211:31–6. 10.1192/bjp.bp.116.192799 28673946 PMC5494438

[26] Linden S Rich C. The use of lithium during pregnancy and lactation. *J Clin Psychiatry.* (1983) 44:358–61. 6358200

[27] Brodtkorb E Reimers A. Seizure control and pharmacokinetics of antiepileptic drugs in pregnant women with epilepsy. *Seizure.* (2008) 17:160–5. 10.1016/j.seizure.2007.11.015 18158256

[28] Iorga A Horowitz B. *Phenytoin Toxicity.* Treasure Island (FL): StatPearls Publishing LLC (2022).

[29] Chen S Perucca E Lee J Richens A. Serum protein binding and free concentration of phenytoin and phenobarbitone in pregnancy. *Br J Clin Pharmacol.* (1982) 13:547–52. 10.1111/j.1365-2125.1982.tb01420.x 7066170 PMC1402062

[30] Isoherranen N Thummel K. Drug metabolism and transport during pregnancy: how does drug disposition change during pregnancy and what are the mechanisms that cause such changes? *Drug Metab Dispos.* (2013) 41:256–62. 10.1124/dmd.112.050245 23328895 PMC3558867

[31] Peng J Ladumor M Unadkat J. Prediction of pregnancy-induced changes in secretory and total renal clearance of drugs transported by organic anion transporters. *Drug Metab Dispos.* (2021) 49:929–37. 10.1124/dmd.121.000557 34315779 PMC8626639

[32] Eyal S Easterling T Carr D Umans J Miodovnik M Hankins G Pharmacokinetics of metformin during pregnancy. *Drug Metab Dispos.* (2010) 38:833–40. 10.1124/dmd.109.031245 20118196 PMC2872944

[33] Shiu J Ensom M. Dosing and monitoring of methadone in pregnancy: literature review. *Can J Hosp Pharm.* (2012) 65:380–6. 10.4212/cjhp.v65i5.1176 23129867 PMC3477836

[34] Daud A Bergman J Bakker M Wang H Kerstjens-Frederikse W de Walle H P-Glycoprotein-mediated drug interactions in pregnancy and changes in the risk of congenital anomalies: a case-reference study. *Drug Saf.* (2015) 38:651–9. 10.1007/s40264-015-0299-3 26017034 PMC4486783

[35] Eke A Mirochnick M. Ritonavir and cobicistat as pharmacokinetic enhancers in pregnant women. *Expert Opin Drug Metab Toxicol.* (2019) 15:523–5. 10.1080/17425255.2019.1628947 31185758 PMC9210948

[36] Eke A Mirochnick M. Cobicistat as a pharmacoenhancer in pregnancy and postpartum: progress to date and next steps. *J Clin Pharmacol.* (2019) 59:779–83. 10.1002/jcph.1397 30821843 PMC9228990

[37] Paxil CR. *Paxil (Paroxetine Hydrochloride) [package insert].* Research Triangle Park, NC: GlaxoSmithKline (2010).

[38] Koren G Matsui D Einarson A Knoppert D Steiner M. Is maternal use of selective serotonin reuptake inhibitors in the third trimester of pregnancy harmful to neonates? *Cmaj.* (2005) 172:1457–9. 10.1503/cmaj.1041100 15911861 PMC557982

[39] Warburton W Hertzman C Oberlander TF. A register study of the impact of stopping third trimester selective serotonin reuptake inhibitor exposure on neonatal health. *Acta Psychiatr Scand.* (2010) 121:471–9. 10.1111/j.1600-0447.2009.01490.x 19878137

[40] Paxil CR. *Paxil (Paroxetine Hydrochloride) [Package Insert].* Research Triangle Park, NC: GlaxoSmithKline (2014).

[41] Westin A Brekke M Molden E Skogvoll E Spigset O. Selective serotonin reuptake inhibitors and venlafaxine in pregnancy: changes in drug disposition. *PLoS One.* (2017) 12:e0181082. 10.1371/journal.pone.0181082 28708853 PMC5510868

[42] Brent G. Maternal hypothyroidism: recognition and management. *Thyroid.* (1999) 9:661–5. 10.1089/thy.1999.9.661 10447011

[43] Dash S Sahoo N Rout U Mishra S Swain J Mazumder A. Outcomes with levothyroxine treatment in early pregnancy with subclinical hypothyroidism. *Cureus.* (2022) 14:e24984. 10.7759/cureus.24984 35719785 PMC9191263

[44] Moog N Entringer S Heim C Wadhwa P Kathmann N Buss C. Influence of maternal thyroid hormones during gestation on fetal brain development. *Neuroscience.* (2017) 342:68–100. 10.1016/j.neuroscience.2015.09.070 26434624 PMC4819012

[45] Nooney J Thor S de Vries C Clements J Sahin L Hua W Assuring access to safe medicines in pregnancy and breastfeeding. *Clin Pharmacol Ther.* (2021) 110:941–5. 10.1002/cpt.2212 33615448 PMC8518426

